# Individual Contributions of Cardiac Ion Channels on Atrial Repolarization and Reentrant Waves: A Multiscale In-Silico Study

**DOI:** 10.3390/jcdd9010028

**Published:** 2022-01-14

**Authors:** Henry Sutanto

**Affiliations:** 1 Department of Physiology and Pharmacology, State University of New York (SUNY) Downstate Health Sciences University, Brooklyn, NY 11203, USA; henry1988md@gmail.com; 2 Department of Cardiology, CARIM School for Cardiovascular Diseases, Maastricht University, 6229 ER Maastricht, The Netherlands

**Keywords:** cardiovascular physiology, cardiac cellular electrophysiology, atrial ion channel, arrhythmia, multiscale computational modeling

## Abstract

The excitation, contraction, and relaxation of an atrial cardiomyocyte are maintained by the activation and inactivation of numerous cardiac ion channels. Their collaborative efforts cause time-dependent changes of membrane potential, generating an action potential (AP), which is a surrogate marker of atrial arrhythmias. Recently, computational models of atrial electrophysiology emerged as a modality to investigate arrhythmia mechanisms and to predict the outcome of antiarrhythmic therapies. However, the individual contribution of atrial ion channels on atrial action potential and reentrant arrhythmia is not yet fully understood. Thus, in this multiscale in-silico study, perturbations of individual atrial ionic currents (I_Na_, I_to_, I_CaL_, I_Kur_, I_Kr_, I_Ks_, I_K1_, I_NCX_ and I_NaK_) in two in-silico models of human atrial cardiomyocyte (i.e., Courtemanche-1998 and Grandi-2011) were performed at both cellular and tissue levels. The results show that the inhibition of I_CaL_ and I_NCX_ resulted in AP shortening, while the inhibition of I_Kur_, I_Kr_, I_Ks_, I_K1_ and I_NaK_ prolonged AP duration (APD). Particularly, in-silico perturbations (inhibition and upregulation) of I_Kr_ and I_Ks_ only minorly affected atrial repolarization in the Grandi model. In contrast, in the Courtemanche model, the inhibition of I_Kr_ and I_Ks_ significantly prolonged APD and vice versa. Additionally, a 50% reduction of I_to_ density abbreviated APD in the Courtemanche model, while the same perturbation prolonged APD in the Grandi model. Similarly, a strong model dependence was also observed at tissue scale, with an observable I_K1_-mediated reentry stabilizing effect in the Courtemanche model but not in the Grandi atrial model. Moreover, the Grandi model was highly sensitive to a change on intracellular Ca^2+^ concentration, promoting a repolarization failure in I_CaL_ upregulation above 150% and facilitating reentrant spiral waves stabilization by I_CaL_ inhibition. Finally, by incorporating the previously published atrial fibrillation (AF)-associated ionic remodeling in the Courtemanche atrial model, in-silico modeling revealed the antiarrhythmic effect of I_Kr_ inhibition in both acute and chronic settings. Overall, our multiscale computational study highlights the strong model-dependent effects of ionic perturbations which could affect the model’s accuracy, interpretability, and prediction. This observation also suggests the need for a careful selection of in-silico models of atrial electrophysiology to achieve specific research aims.

## 1. Introduction

The cardiac action potential (AP) results from a complex dynamic behavior of ionic currents within a cardiomyocyte. Together, they maintain the excitation, contraction, and relaxation of a cardiomyocyte. In a physiological condition, the activation of voltage-gated Na^+^ channels induces the opening of other voltage-gated ion channels, including the L-type Ca^2+^ channels, allowing access of Ca^2+^ from the extracellular space to the cytoplasm. Subsequently, the Ca^2+^ influx initiates Ca^2+^-induced Ca^2+^ release (CICR), releasing more Ca^2+^ from the sarcoplasmic reticulum to the cytosolic space to exert their function in cardiomyocyte contraction and other Ca^2+^-dependent signaling processes [1]. Such an activation of fast Na^+^ channels is denoted as a positive deflection (rapid depolarization; phase 0) of the cardiac AP, whereas the opening of the L-type Ca^2+^ channels modulate the plateau phase (phase 2) of the AP. During repolarization, several K^+^ channels, including the transient-outward and delayed- and inward-rectifier K^+^ channels, are activated. In the atria, some atrial-specific K^+^ channels (e.g., Ca^2+^-activated, two-pore domain and ultra-rapid delayed-rectifier K^+^ channels) have also been reported to hold an important role in the AP repolarization. Such a repolarization is displayed as a negative deflection of the AP (phase 1 and 3), restoring the membrane potential to the resting state (phase 4). Meanwhile, in the presence of disease-associated ionic remodeling, the collaborative efforts between ion channels could be disrupted, allowing cardiac arrhythmias to occur and persist. Therefore, AP morphology and AP duration (APD) are commonly considered as cellular markers of cardiac arrhythmias.

Computational modeling of atrial electrophysiology has been employed to study the mechanisms of atrial arrhythmias, to predict the outcome of antiarrhythmic interventions, and to assist decision-making process in the clinic [2,3]. For example, multiscale computational modeling was employed to study the ethanol-associated atrial arrhythmogenesis [4] and to support individualized planning for catheter ablation procedures of atrial arrhythmias [5]. However, the distinct formulations within each atrial model could affect the simulated results and therefore might influence the accuracy of in-silico models to predict the outcome of specific interventions. Moreover, a better understanding on the individual contribution of atrial ion channels on atrial repolarization is a prerequisite for designing an appropriate channel-targeted antiarrhythmic therapy.

Therefore, this multiscale in-silico study sought to explore the individual contribution of atrial ionic currents on AP repolarization and reentrant waves behavior in two distinct in-silico models of atrial cellular electrophysiology, and to test several potential antiarrhythmic strategies to destabilize AF-associated reentrant spiral waves in silico.

## 2. Materials and Methods

### 2.1. Zero-Dimensional Computational Modeling

The individual contribution of nine major ionic currents (fast Na^+^ current [I_Na_], transient-outward K^+^ current [I_to_], L-type Ca^2+^ current [I_CaL_], ultra-rapid delayed-rectifier K^+^ current [I_Kur_], rapid delayed-rectifier K^+^ current [I_Kr_], slow delayed-rectifier K^+^ current [I_Ks_], inward-rectifier K^+^ current [I_K1_], Na^+^-Ca^2+^ exchange current [I_NCX_] and Na^+^-K^+^ pump current [I_NaK_]) were assessed in two in-silico human atrial cardiomyocyte models, namely Courtemanche et al. [6] and Grandi et al. [7]. To simulate the cellular effect of AF-associated ionic remodeling, the AF variant of the Courtemanche model, which was made by incorporating the chronic-AF-associated electrical remodeling of transmembrane currents reported by Grandi et al. [7] (i.e., I_Na_ −10%, I_to_ −80%, I_CaL_ −50%, I_Kur_ −55%, I_Ks_ +100%, I_K1_ +100% and I_NCX_ +40%) were employed. AP simulations were performed at a 1 Hz pacing frequency (basic cycle length [BCL] of 1000 ms) and quasi-steady-state APD for the baseline models was obtained following 100 beats of pre-pacing. All original models were obtained from CellML and all simulations were performed in Myokit [8].

### 2.2. Two-Dimensional Computational Modeling

Reentrant spiral waves were simulated using an S_1_S_2_ induction protocol in homogeneous tissue of 4 × 4 cm (200 × 200 units) following 100 beats of 1 Hz pre-pacing at the cellular level. The tissue CV was maintained around 45–50 cm/s for the baseline models and allowed to vary in the presence of underlying ionic perturbations. The first stimulus (S_1_) was initiated from left to right to generate a normal excitation wave. Subsequently, the second stimulus (S_2_) was applied to the upper-left quadrant of the tissue, generating an additional wavefront that can interact with the tail of the preceding wave, producing reentry in a vulnerable substrate [4]. The vulnerable window was evaluated to assess both the inducibility and stability of reentrant arrhythmias under different conditions. The size of the vulnerable window indicates the inducibility of reentry, whereas the duration of reentry within the vulnerable window indicates the stability of reentrant arrhythmias. A stable reentry was defined as a reentrant spiral wave that persisted after 12 s.

## 3. Results

### 3.1. Individual Contribution of Ion Channels on Atrial Action Potential Repolarization

**Figure 1:**
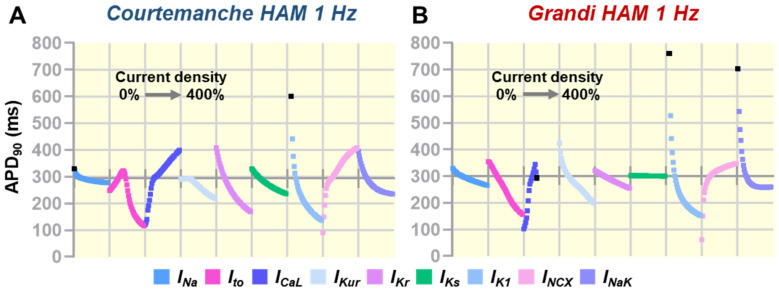
(**A**,**B**) The effect of G_max_ perturbations (0% to 400%) on APD_90_ between models. Each segment on the horizontal axes corresponds to a range of perturbations. Traces were discontinued (marked as black squares) when no APD_90_ could be measured (e.g., repolarization failure/plateau arrest). The position of the horizontal axes indicates the baseline APD_90_ for each model (100% G_max_).

**Figure 2:**
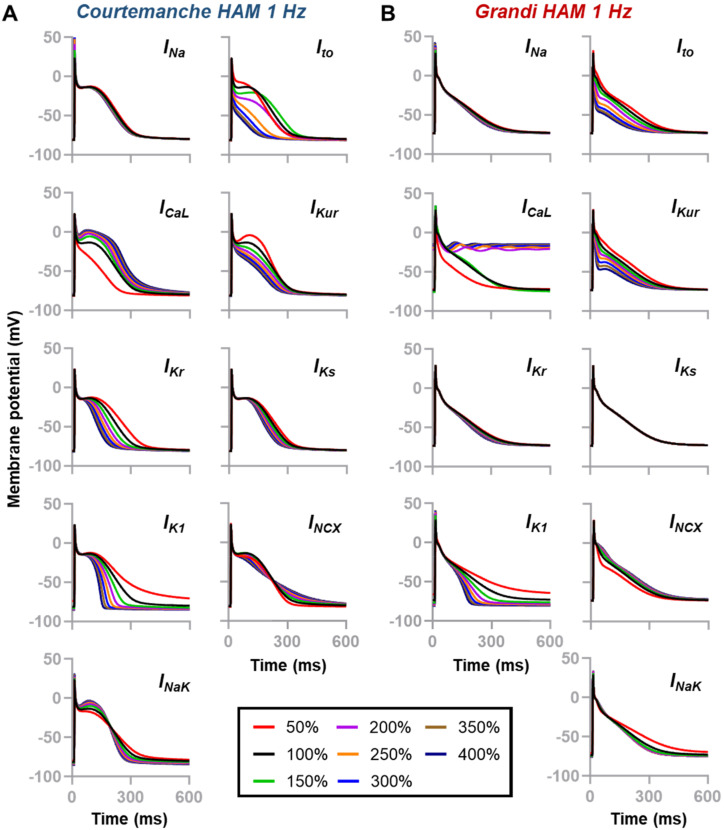
(**A**,**B**) Individual contribution of ionic currents on the repolarization of human atrial cardiomyocytes. Two human atrial cardiomyocyte models [Courtemanche et al. [6] and Grandi et al. [7]] were used to assess the contribution of nine major ionic currents (i.e., I_Na_, I_to_, I_CaL_, I_Kur_, I_Kr_, I_Ks_, I_K1_, I_NCX_ and I_NaK_) on atrial repolarization. Channel maximum conductance (G_max_) were scaled from 50% to 400% of the default value.

To explore the contribution of individual atrial ionic currents on AP repolarization, a sensitivity analysis was conducted by varying the maximum conductance (G_max_) of ionic currents of interest up to 400% of the default values (Figure 1 and Figure 2). Figure 1 display sensitivity plots describing the changes of APD at 90% repolarization (APD_90_) due to ionic current alterations (0% to 400%). In the Courtemanche model (Figure 1A and Figure 2A), all currents, except I_Na_, had a meaningful effect on the AP repolarization phase, whereas in the Grandi human atrial cardiomyocyte model (Figure 1B and Figure 2B), I_Kr_ and I_Ks_ had limited contribution on AP repolarization. Notably, a 50% reduction of I_to_ abbreviated the APD_90_ in the Courtemanche model (268 ms vs. 293 ms [−8.5%]), whereas APD_90_ was prolonged (331 ms vs. 302 ms [+9.6%]) in the Grandi model. The Grandi model also exhibited a high sensitivity to I_CaL_ perturbations, with I_CaL_ above 150% of the baseline model resulting in plateau arrest (Figure 2B). Meanwhile, the alteration of I_Kur_ affected the entire phase of repolarization in the Grandi model, but its effect was primarily in the early phase of repolarization in the Courtemanche model. In both atrial models, I_K1_ perturbations notably affected the late phase of repolarization and resting membrane potential (RMP), strongly modulating APD_90_. In the Courtemanche model, perturbations in I_NCX_ and I_NaK_ that prolonged APD during the early repolarization phase shortened APD_90_.

### 3.2. The Effects of Ionic Current Conductance Perturbation on Reentrant Spiral Waves

The effect of an antiarrhythmic therapy in the cellular level could be different than that in the tissue and organ levels due to the potential influence of cell-cell interactions. Therefore, to improve our understanding on the consequences of ion-channel perturbations on the behavior of reentrant arrhythmias, we performed sensitivity analyses in homogenous atrial tissues, assessing the individual contribution of nine major ionic currents.

**Figure 3:**
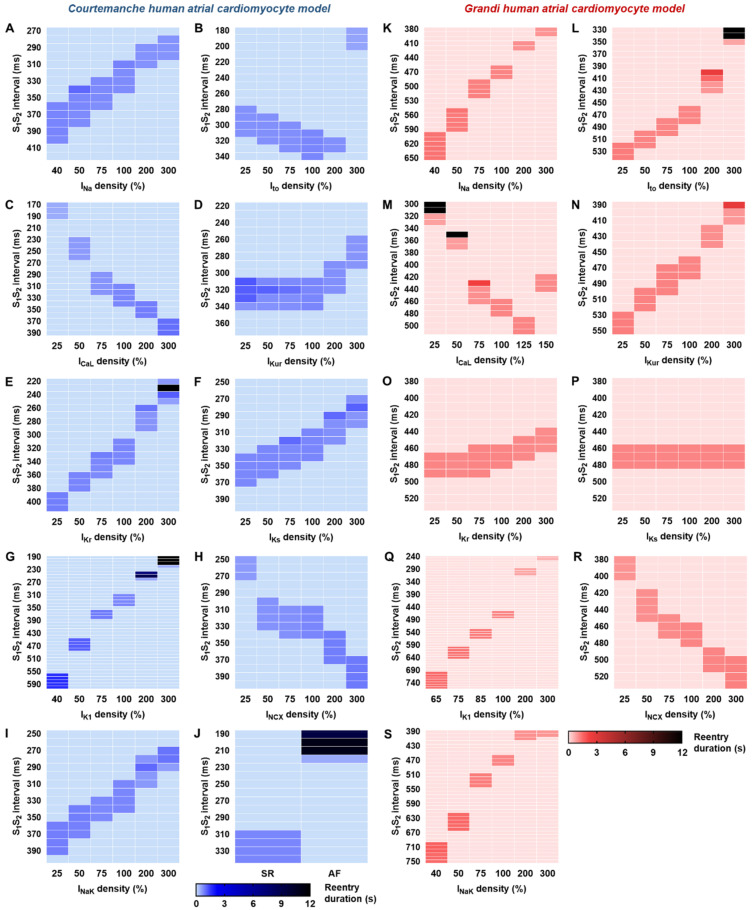
Individual contributions of atrial ionic currents on reentrant waves behavior. Vulnerable windows depicting the effects of perturbations of nine atrial ionic currents on spiral waves generation, maintenance and termination in the Courtemanche (**A**–**I**) and Grandi human atrial cardiomyocyte (**K**–**S**) models. (**J**) The effect of previously documented AF-related ion-channel remodeling [7] on cardiac reentry.

Inhibitions of I_Na_ shifted the vulnerable windows to larger S_1_S_2_ intervals and widened the size of the vulnerable windows, indicating a higher inducibility of re-entry in both the Courtemanche and Grandi models (Figure 3A,K). Similar to the cellular sensitivity analysis (Figure 2), the response to I_to_ block was different in the Courtemanche and Grandi models. In the Courtemanche tissue model (Figure 3B), I_to_ block produced a shift of the vulnerable windows to the earlier S_1_S_2_ intervals, without affecting the reentry inducibility. In contrast, in the Grandi model (Figure 3L), I_to_ block shifted the vulnerable windows to larger S_1_S_2_ intervals, consistent with the cellular APD response. The upregulation of I_to_ up to 200% in the Courtemanche model reduced the inducibility of reentrant waves, whereas increasing I_to_ to 300% markedly shifted the windows to earlier S_1_S_2_ intervals. However, the stability of reentry was not affected by I_to_ perturbations. Meanwhile, increasing I_to_ in the Grandi model shifted the vulnerable windows to earlier S_1_S_2_ intervals. At 200% I_to_, the stability of reentry increased, and a stable reentry was attained with 300% I_to_.

Reductions in I_CaL_ shifted the vulnerable windows to earlier S_1_S_2_ intervals in both models, with some instances of stable reentry in the Grandi model following 25–50% I_CaL_ (Figure 3M), whereas I_CaL_ upregulation shifted the windows to larger S_1_S_2_ intervals in the Courtemanche model (Figure 3C) and to shorter intervals in the Grandi model (Figure 3M). Of note, the I_CaL_ upregulation cannot exceed 150% in the Grandi model due to repolarization failure at higher current densities. Alterations of I_Kr_ and I_Ks_ did not have a major effect on AP properties in the Grandi model (Figure 2). Similarly, at the tissue level, only I_Kr_ upregulation shifted the vulnerable windows to earlier S_1_S_2_ intervals, with no effect on the inducibility and stability of reentry (Figure 3O). Meanwhile, in the Courtemanche model, I_Kr_ upregulation (300%) stabilized reentrant waves (Figure 3E). Similarly, the upregulation of I_K1_ could stabilize reentrant arrhythmias in the Courtemanche model (Figure 3G), while no stable reentry was documented in the Grandi model (Figure 3Q). Figure 3J shows the consequence of AF-related electrical remodeling incorporated in the Courtemanche atrial model on the behavior of atrial reentry.

**Figure 4:**
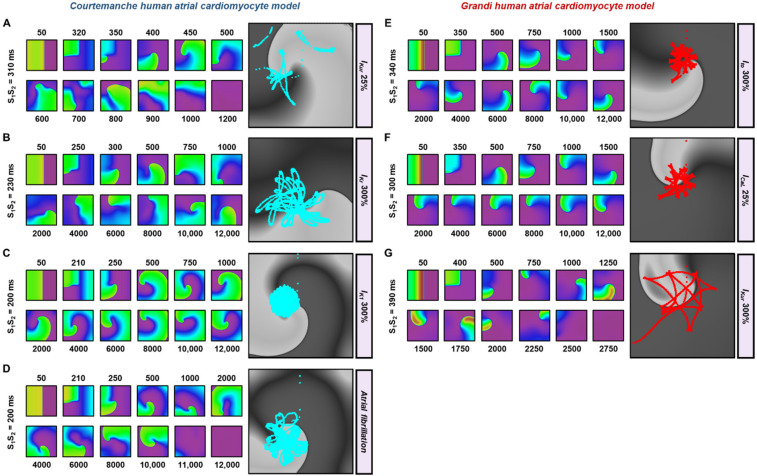
Example snapshots of 2-dimensional tissue simulations showing the effects of ionic current alteration on reentrant waves. (**A**–**D**) The effects of ionic current perturbations (25% I_Kur_, 300% I_Kr_, 300% I_K1_ and AF electrical remodeling) on reentry in the Courtemanche human atrial cardiomyocyte model. (**E**–**G**) The effects of ionic current alterations (300% I_to_, 25% I_CaL_ and 300% I_Kur_) on reentry in the Grandi human atrial cardiomyocyte model. The right panels show the rotor-core trajectories tracked up to 3000 ms.

Figure 4 shows some examples of the effect of ion-channel perturbations on reentrant spiral waves. As indicated above, 300% I_Kr_, 300% I_K1_ and AF electrical remodeling triggered stable reentry in the Courtemanche model, whereas 300% I_to_ and 25% I_CaL_ resulted in similar behavior in the Grandi model. The rotor cores (Figure 4A–H, right panels) meandered less in the stable reentry as compared to the unstable ones (Figure 4A,G), consistent with previous work [9]. Overall, our tissue-scale sensitivity analyses, as previously presented [10], indicate a strong model dependence of the effect of ionic perturbations on reentrant arrhythmias, making it difficult to interpret their exact consequences in silico. These findings emphasize the importance of developing a personalized in-silico model to assess the patient-specific arrhythmogenic risk and predict the individual response to antiarrhythmic therapies.

### 3.3. In-Silico Assessment of Antiarrhythmic Properties of Ionic Current Perturbations

**Figure 5:**
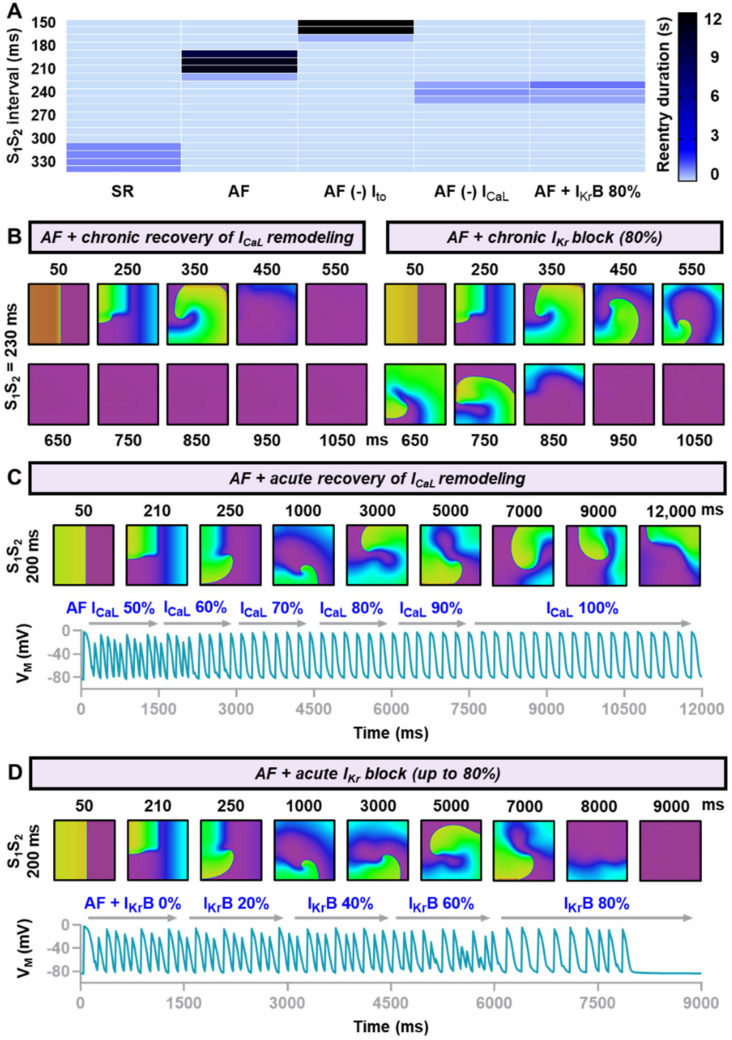
Chronic and acute effects of hypothetical treatment to restore the function of altered ion channels. (**A**) Vulnerable windows of the Courtemanche model comparing reentrant waves behavior under sinus rhythm (SR), atrial fibrillation (AF), AF without I_to_ remodeling, AF without I_CaL_ remodeling and AF with 80% I_Kr_ block, mimicking the effect of class III antiarrhythmic drugs. (**B**) Chronic restoration of I_CaL_ and chronic block of I_Kr_ altered the stability of reentry, resulted in the abbreviation of reentry duration. (**C**,**D**) The acute effects of I_CaL_ restoration and I_Kr_ block on cardiac reentry.

Taking into account the insight from the tissue sensitivity analyses, we tested the efficacy of three different modalities to treat AF-associated electrical remodeling in homogenous atrial tissues using the Courtemanche human atrial cardiomyocyte model: the recovery of AF-related I_to_ remodeling, recovery of AF-related I_CaL_ remodeling and 80% I_Kr_ block, mimicking the effect of class III antiarrhythmic drugs (e.g., dofetilide and ibutilide). Reversing I_to_ remodeling shifted the vulnerable windows to the earlier S_1_S_2_ intervals and slightly reduced the inducibility of reentrant waves. However, some episodes of stable reentry were still detected (Figure 5A). On the contrary, reversing I_CaL_ remodeling shifted the vulnerable windows to the larger S_1_S_2_ intervals and destabilized reentry, with no stable reentry remained. Similarly, 80% block of I_Kr_ also countered the AF electrical remodeling and yielded similar effects to I_CaL_ remodeling recovery.

Based on these findings, we further explored whether I_CaL_ recovery and I_Kr_ block had identical antiarrhythmic properties in the virtual atrial tissue. To achieve this aim, we tested the efficacy of both interventions in two different circumstances, namely acute and chronic settings. In an acute setting, the pharmacological intervention is applied after the reentry is initiated. Meanwhile, in the chronic setting, the treatment modality is applied before the reentry occurs. Clinically, the former resembles an acute treatment of cardiac arrhythmias, whereas the latter mimics the ability of a treatment modality to prevent the reoccurrence or exacerbation of cardiac arrhythmias. Figure 5B depicted the representative snapshots of reentrant waves (S_1_S_2_ interval = 230 ms) demonstrating that both modalities were effective in destabilizing reentrant arrhythmias in the chronic setting. However, Figure 5C,D clearly showed that only 80% I_Kr_ block was effective to destabilize and terminate reentrant arrhythmias in an acute setting, whereas acute recovery of I_CaL_ remodeling preserved the existence of the reentrant spiral wave.

## 4. Discussion

The repolarization of atrial action potential is maintained by numerous ionic currents [11,12,13,14,15,16,17]. In canine atrium preparations, lidocaine (a class IB antiarrhythmic drug that predominantly blocks I_Na_) minimally shortened APD_90_ but caused a significant effective refractory period (ERP) prolongation. Meanwhile, E-4031 (a specific I_Kr_ blocker) prolonged both APD_90_ and ERP, whereas the combination of the two displayed a synergistic AF-suppressing effect [18]. The effects of I_Na_ and I_Kr_ inhibition on atrial cardiomyocytes were also seen in our study, in which I_Na_ inhibitions had negligible impact on cellular APD_90_ (Figure 2) but significantly shifted the vulnerable windows to the larger S_1_S_2_ intervals (Figure 3), suggesting a prolongation of tissue ERP. Additionally, the reduction of I_Kr_ extended the APD_90_ and shifted the vulnerable windows to the larger S_1_S_2_ intervals in the Courtemanche human atrial model. Several studies have also highlighted the importance of I_to_ in the repolarization of atrial cardiomyocytes. A human right atrial experiment revealed that I_to_ produced pronounced changes on early repolarization of atrial AP and force generation [19], and its contribution was effective at all physiological heart rates in humans [20]. Ni et al. [21] also explored the contributions of I_to_ in silico and demonstrated that I_to_ modulated the atrial APD restitution and its alteration contributed to APD alternans. The in-silico effect of I_to_ upregulation on APD seemed to be consistent across computational models of atrial cardiomyocytes [17], including our current work, in which we observed reductions of APD in both early and late repolarization. At tissue scale, the role of I_K1_ in reentrant wave stabilization has been documented in both computational and experimental studies [22]. Computer simulations indicated that in addition to the APD shortening, I_K1_ upregulation also increased the availability of I_Na_, which further accelerated the rotor. Experimentally, a similar observation was documented in I_K1_-overexpressed mice, in which the rotor was persistent and faster than the wildtype animals [22].

Consistent with our simulation in Figure 5D, in addition to its effect on the prevention of AF recurrence, dofetilide has been widely used as an effective rhythm-control strategy for acute AF cardioversion. Spontaneous conversion of persistent AF was commonly seen within three days of dofetilide administration [23]. Meanwhile, although currently no available pharmacological agent could revert the pre-existed AF-associated I_CaL_ remodeling, in the future, a drug that upregulates the I_CaL_ density, possibly through an alteration of channel’s kinetics or expression, could be beneficial to prevent the AF recurrence, as we demonstrated previously in Figure 5A,B. Alternatively, gene therapy might also be useful to revert AF remodeling through several ways, as previously discussed by Liu and Donahue [24].

Next, our multiscale computational study also indicates strong model-dependent effects of ionic perturbations on atrial electrophysiology. For example, in the Grandi atrial model, the contributions of I_Kr_ and I_Ks_ on atrial repolarization and reentrant waves behavior were minor, affecting the suitability of this model for in-silico drug cardiotoxicity screening and the mechanistic investigation on the arrhythmic consequences of disease-associated I_Kr_ and I_Ks_ remodeling. Of note, experimentally, the presence of both rapid and slow components of delayed-rectifier K^+^ (Kr and Ks) channels has been previously reported in human atrial cardiomyocytes isolated from right atrial appendages extracted at the time of coronary artery bypass surgery [25]. Additionally, in Figure 2 and Figure 3, we also exemplified the model-dependent effect of I_to_ inhibition on atrial AP and reentrant waves. In the Grandi human atrial cardiomyocyte model, 50% I_to_ inhibition prolonged APD and shifted the vulnerable windows to larger S_1_S_2_ intervals, whereas in the Courtemanche human atrial model, I_to_ inhibition resulted in the shortening of APD_90_ and the shifting of vulnerable windows to earlier S_1_S_2_ intervals. Experimentally, the inhibition of I_to_ by 4-aminopyridine (4-AP) in human atrial cardiomyocytes obtained from human right atrial appendages extracted at the time of bypass surgery also abbreviated the phase 1 of AP (increasing the plateau height) and shortened APD_90_ without any effect on RMP [19], consistent with our observation in the Courtemanche human atrial model (Figure 1A).

Overall, the agreement of our findings with previous experimental studies demonstrates that in-silico modeling is a powerful and robust tool to study the mechanistic background of atrial arrhythmias (e.g., to explore the effects of ion-channel perturbation on atrial AP and reentrant arrhythmias) and to guide arrhythmia therapy in the clinic (e.g., to assess the acute and chronic effects of anti-AF medications). Nonetheless, a careful assessment on the appropriateness of each mathematical model to do specific tasks is warranted due to the strong model dependence. Norbert Wiener’s infamous quote in 1945—“*The best material model for a cat is another, or preferably the same cat*” highlights such a basic limitation of computational models that no in-silico model could perfectly match the modeled biological systems (e.g., cells, tissues or organ). Also, it is important to note that an in-silico model is commonly validated to a certain extent and is designed to fulfil specific research objectives, therefore the application beyond those circumstances (i.e., the validated range of operation) requires careful consideration [26].

Finally, several limitations of this study need to be acknowledged. First, when simulating the AF-related remodeling, we did not incorporate the AF-associated remodeling of Ca^2+^-handling proteins, as well as AF-induced structural remodeling. Second, we also did not incorporate atrial-specific ion channels in the models since they are not available in the original models and for acetylcholine-activated inward-rectifying K^+^ currents, since we did not simulate the effects of parasympathetic/vagal response in this study. Third, we performed the tissue simulations in a small sized tissue (4 × 4 cm) due to limited computational power. Our preliminary assessment (Supplemental Figure S1) revealed that tissue size could influence the stability of reentry, presumably due to the availability of excitable area for wave propagation, allowing the meandering rotor to persist. However, we speculate that this issue would not affect the overall results of this study since all of the comparisons were made in the same tissue size, although the confirmation of this notion in a larger tissue size is warranted. Additionally, in the future, an extension to organ-level simulations could be performed.

## 5. Conclusions

Atrial repolarization is regulated by multiple ion channels, which further modulate the behavior of reentrant spiral waves in the tissue level. Our multiscale computational study highlights the strong model-dependent effects of ionic perturbations which could affect the model’s accuracy, interpretability, and prediction. This observation also suggests the need for a careful selection of in-silico models of atrial electrophysiology to achieve specific research aims.

## Data Availability

Computer codes used in this study, such as the Myokit files for the zero- and two-dimensional simulations, are available from: https://github.com/henrysutanto (accessed on 2 December 2021).

## Supplementary Materials

The following are available online at https://www.mdpi.com/article/10.3390/jcdd9010028/s1. Supplemental Figure S1: The comparison of reentrant waves in two different virtual tissue sizes.
