# Supplementary material for: Individual Contributions of Cardiac Ion Channels on Atrial Repolarization and Reentrant Waves: A Multiscale In-Silico Study
Source: J Cardiovasc Dev Dis. 2022 Jan 14;9(1):28. doi: 10.3390/jcdd9010028 (PMC8779488; doi:10.3390/jcdd9010028)
Supplement: Supplementary file 1 [file jcdd-09-00028-s001.zip › jcdd-1516850-supplementary.pdf]

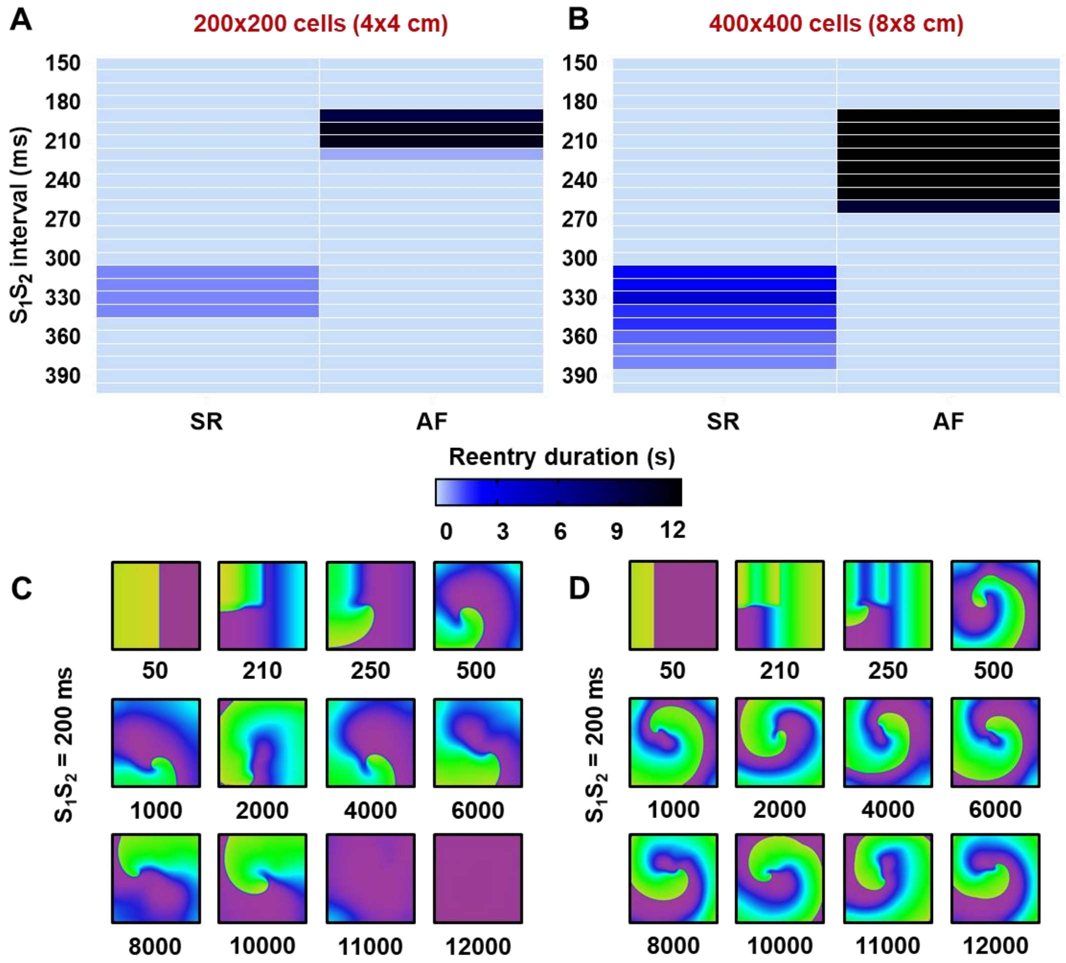

Supplemental Figure S1. The comparison of reentrant waves in two different virtual tissue sizes. A) The vulnerable window of sinus rhythm (SR) and atrial fibrillation (AF) in the Courtemanche human atrial model simulated in a 4x4 cm of tissue (200x200 cells). **B)** The vulnerable window of SR and AF in the Courtemanche human atrial model simulated in an 8x8 cm of tissue (400x400 cells). **C-D)** Snapshots of the reentrant wave at the S_1S_2 interval of 200 ms. In both tissues, 3 x n of the leftmost cells were paced (n = the size of the tissue) to generate an activation wavefront. Conduction velocity is maintained around 50 cm/s in both tissue sizes.
